# Deep learning model for automatic image quality assessment in PET

**DOI:** 10.1186/s12880-023-01017-2

**Published:** 2023-06-05

**Authors:** Haiqiong Zhang, Yu Liu, Yanmei Wang, Yanru Ma, Na Niu, Hongli Jing, Li Huo

**Affiliations:** 1 Department of Nuclear Medicine, State Key Laboratory of Complex Severe and Rare Diseases, Beijing Key Laboratory of Molecular Targeted Diagnosis and Therapy in Nuclear Medicine, Peking Union Medical College Hospital, Chinese Academy of Medical Sciences, Beijing, 100730 China; 2 Medical Science Research Center, Peking Union Medical College Hospital, Chinese Academy of Medical Sciences, Beijing, 100730 China; 3 GE Healthcare China, Shanghai, 200040 China

**Keywords:** PET, Image quality, Deep learning, Classification

## Abstract

**Background:**

A variety of external factors might seriously degrade PET image quality and lead to inconsistent results. The aim of this study is to explore a potential PET image quality assessment (QA) method with deep learning (DL).

**Methods:**

A total of 89 PET images were acquired from Peking Union Medical College Hospital (PUMCH) in China in this study. Ground-truth quality for images was assessed by two senior radiologists and classified into five grades (grade 1, grade 2, grade 3, grade 4, and grade 5). Grade 5 is the best image quality. After preprocessing, the Dense Convolutional Network (DenseNet) was trained to automatically recognize optimal- and poor-quality PET images. Accuracy (ACC), sensitivity, specificity, receiver operating characteristic curve (ROC), and area under the ROC Curve (AUC) were used to evaluate the diagnostic properties of all models. All indicators of models were assessed using fivefold cross-validation. An image quality QA tool was developed based on our deep learning model. A PET QA report can be automatically obtained after inputting PET images.

**Results:**

Four tasks were generated. Task2 showed worst performance in AUC,ACC, specificity and sensitivity among 4 tasks, and task1 showed unstable performance between training and testing and task3 showed low specificity in both training and testing. Task 4 showed the best diagnostic properties and discriminative performance between poor image quality (grade 1, grade 2) and good quality (grade 3, grade 4, grade 5) images. The automated quality assessment of task 4 showed ACC = 0.77, specificity = 0.71, and sensitivity = 0.83, in the train set; ACC = 0.85, specificity = 0.79, and sensitivity = 0.91, in the test set, respectively. The ROC measuring performance of task 4 had an AUC of 0.86 in the train set and 0.91 in the test set. The image QA tool could output basic information of images, scan and reconstruction parameters, typical instances of PET images, and deep learning score.

**Conclusions:**

This study highlights the feasibility of the assessment of image quality in PET images using a deep learning model, which may assist with accelerating clinical research by reliably assessing image quality.

**Supplementary Information:**

The online version contains supplementary material available at 10.1186/s12880-023-01017-2.

## Background

**Fig.1:**
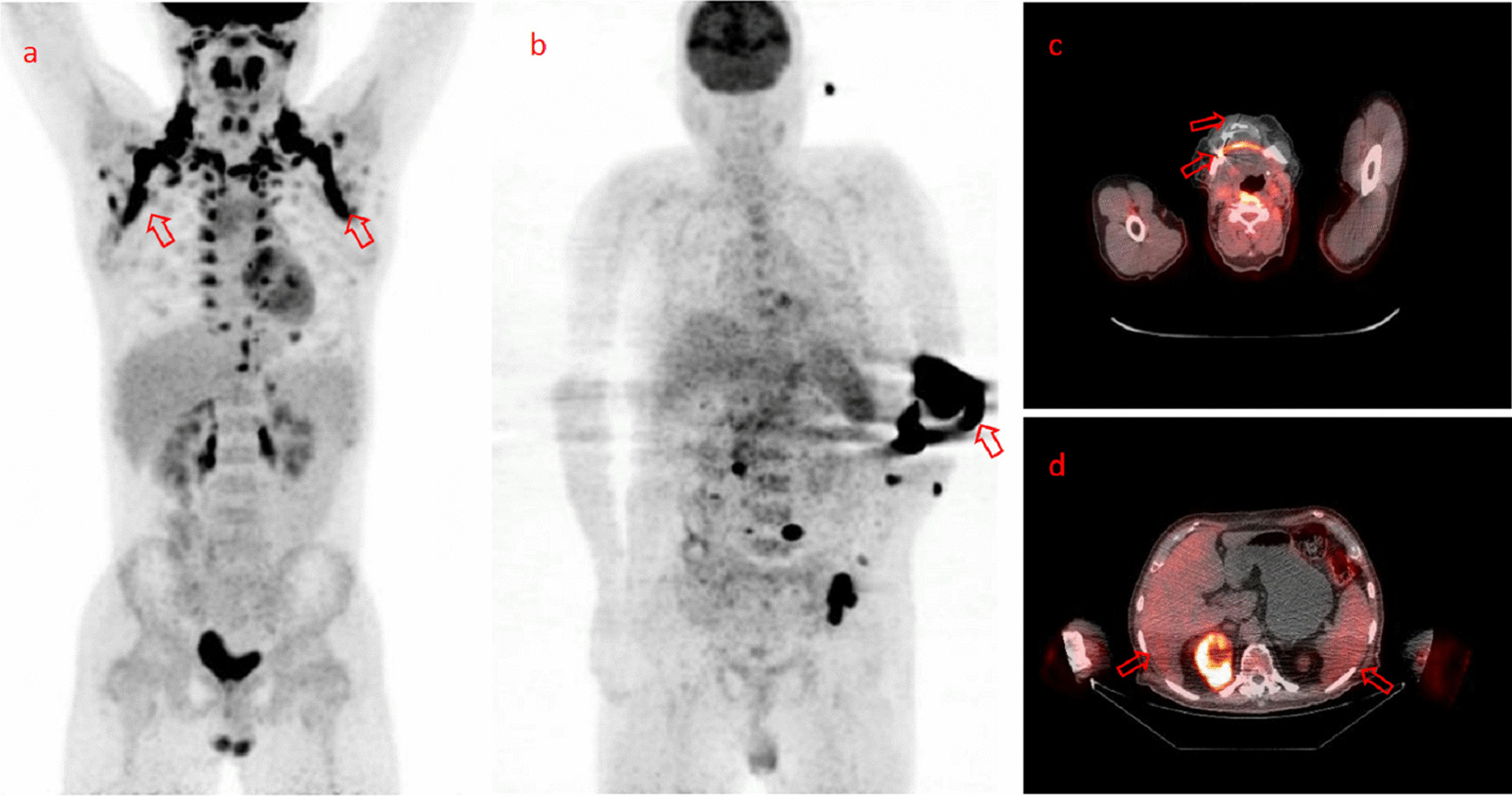
Typical ^18^F-FDG PET/CT images with insufficient level quality: **a** imaging of severe brown fat, **b** intravenous extravasation, **c** mismatch from external patient movement, **d** tissue over-attenuation from improper placement

Molecular imaging of positron emission tomography/computed tomography (PET/CT) has played an important role in nuclear medicine, such as noninvasive tumor diagnostic staging [1], efficacy evaluation [2], and research and development of new drugs [3]. However, high noise levels, missing or incomplete data, motion artifacts, inadequate preparation of patients, intravenous injection failure, improper placement, and scanning equipment miss-calibration might lead to the poor data quality of images and wrong conclusions [4], and the typical images with insufficient level quality are shown in Fig. 1. Therefore, the clinical image quality control of PET/CT is essential for excluding the clinical images with poor quality resulting from any problematic processes and avoiding bias in nuclear medicine medical quality management.

The current traditional clinical image quality evaluations in PET/CT rely on the subjective evaluation with naked eyes following the suggestions in literature [5–7], in which mean liver standardized uptake value normalized by lean body mass (SULs) are expected to be within 1.0 to 2.2 (and mean liver standardized uptake values (SUVs) within 1.3 to 3.0) [5], and blood pool SUL measurements are expected to close 1.2 (and blood pool SUVs around 1.6) [5–7]. However, visual judgment is time-consuming, which is impractical for the evaluation of large-batch images[8]. For artificial quality control, some mistakes in settings of acquisition parameters are easy to be ignored. For example, the statistics are reduced, caused by the shortening of acquisition time [9]. Moreover, there is still a lack of objective and unified standards for artificial visual evaluation, and level differences in doctors can easily lead to evaluation bias. Therefore, a robust, minimally biased, and fully automated PET/CT QA protocol is urgently needed. Convolutional neural networks (CNN) are a good option for the automatic medical image QA domain since they can robustly learn features without knowing a priori.

It is reported that the deep learning CNN technology has been used in different image quality automatically assessment systems, including optical coherence tomography (OCT) images [10], retinal images [11], diabetic retinopathy screening [12], high-frequency ultrasound images [13], CT images [14] and 3D T1-weighted brain MRI images [15]. However, there is still limited research on DL in the automatic control of clinical image quality for PET/CT. In a study, two different CNN algorithms were combined to assess spatial misalignment compared to a standard template, and the signal-to-noise ratio (SNR) difference compared to 200 static quality controlled ^18^F-fluorodopa (FDOPA) PET brain images from three different PET/CT scanners, in which 100% accurate QA classification was reported [16]. However, the ^18^F-FDOPA PET brain images are invalid to the quality control for full-body scans, and ^18^F-FDOPA is at the preclinical stage rather than ^18^F-FDG practical in clinical application. Another preprint shows Elisabeth took EARL standards as a reference and first reported CNN to determine the image quality of a PET torso image, but it is worth mentioning that they utilized only 2D slices instead of 3D volumes of the whole.

The quality evaluation of clinical images in PET/CT is one of the main tasks of the National Nuclear Medicine Quality Control Center in China, where the fully automatic quality control protocol for clinical images in PET/CT is still not available. To solve this problem, we carried out a series of studies on the quality evaluation of PET/CT clinical images. The contributions of this work are summarized as follows. First, the important characteristic parameters were extracted from the original data, and a quality control process was designed. Then, based on the principle of quintuples and the visual judgment of clinical doctors, a deep learning model for automatic quality control for clinical images in PET/CT was constructed, which has the characteristics of multiple parameters generated and large quantities of data required to avoid over-fitting during the training process. This approach pays attention to automated quality assessment based on deep learning, which can automatically assess the quality of PET images, and aims to contribute to the improvement of workflow, better optimization of image acquisition, and enhancing physician efficiency. Finally, combined with the DL-based image quality assessment model, the final QA report will include basic image information, scan and reconstruction parameters, a typical PET imaging example, and deep learning score.

## Methods

### Study design

**Fig.2:**
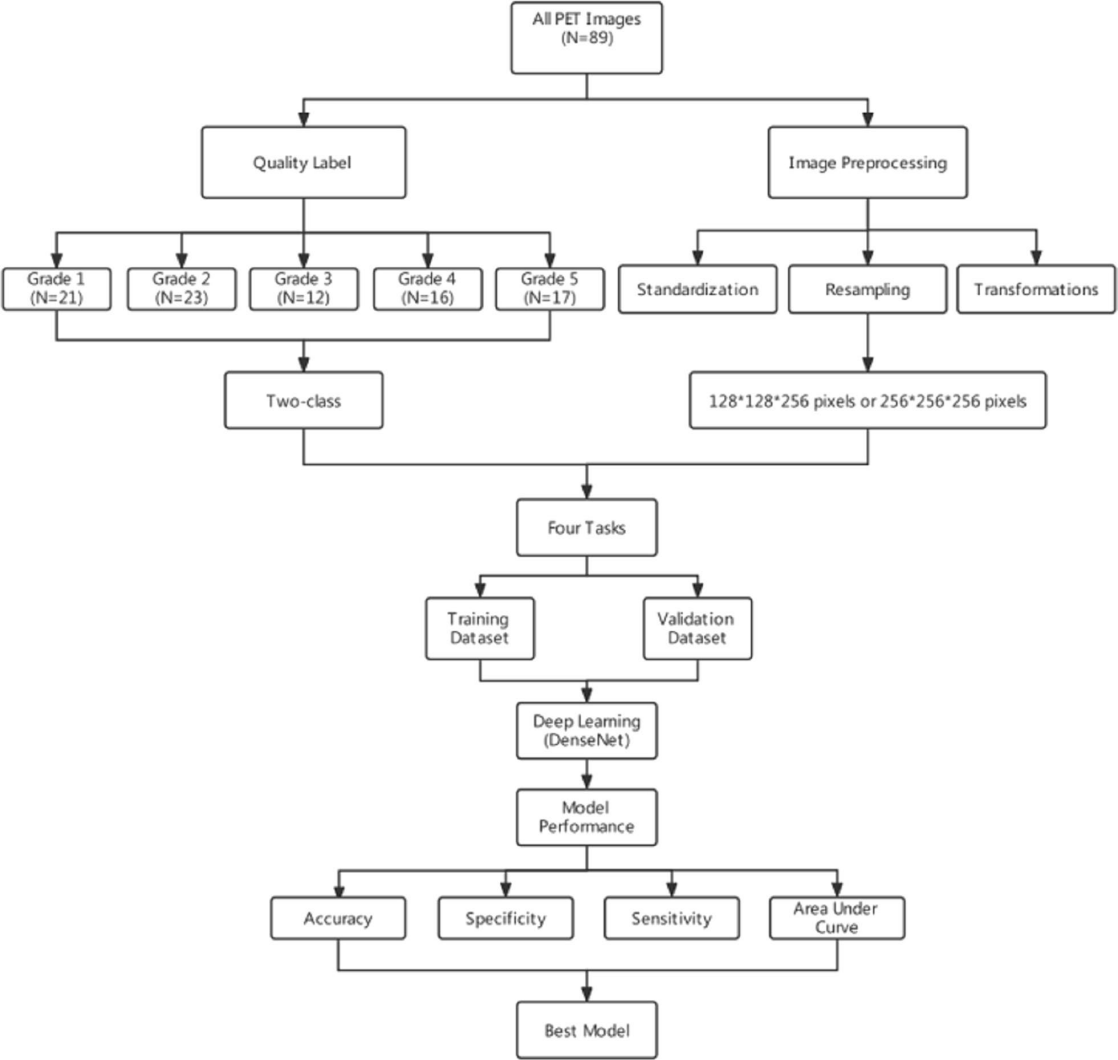
The workflow for this study

The workflow of this study is presented in Fig. 2. This study included four major parts: (i) image acquisition, (ii) image preprocessing, (iii) modal training and cross-validation, and (iv) evaluation.

### Images

In this project, PET images previously obtained during the clinical assessment of various diseases were used to create the datasets utilized for this study. This project contains 89 PET images in clinical routine examination recruited retrospectively from the Peking Union Medical College Hospital (PUMCH) in China. The images were taken using a whole-body PET/CT scanner (Polostar NM680, SinoUnion Healthcare, Beijing, China), and the scanning conditions and parameters are set to be consistent. The PET scanning protocol details were listed as follows. The imaging agent ^18^F-fluorodeoxyglucose (FDG) was produced by PUMCH, with a PH value is about 7.0 and radiochemical purity > 95%. All image acquisition was carried out in a resting state in a quiet and dimly lit room. According to their weight, the patients were given 0.15 mci/kg intravenous injections of ^18^F-FDG for a calm rest for 45–60 min. PET scan used 5–6 beds, 2 min per bed position, and the layer thickness was 5 mm. The PET images were attenuated by CT data and reconstructed by the ordered-subsets expectation maximization (OSEM) algorithm with 10 subsets and 3 iterations, and 4.5-mm full width at half maximum (FWHM) Gaussian post-filtering. The matrix size of all PET reconstructions was 192 × 192, with a pixel size of 3.15 mm × 3.15 mm. Finally, the reconstructed PET images were transmitted to the post-processing platform.

**Fig.3:**
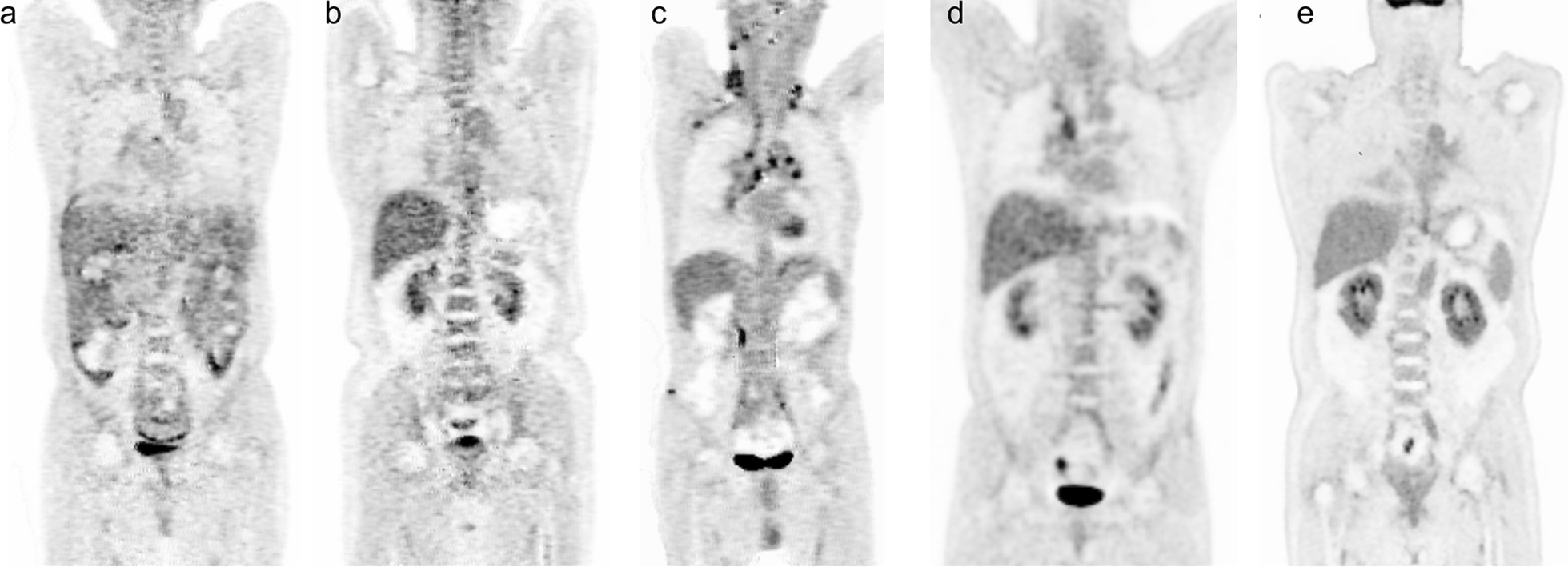
Examples of images with different qualities, including: **a** grade 1, **b** grade 2, **c** grade 3, **d** grade 4, and **e** grade 5

A total of 71 PET images were selected for the training set for this model. The validation set was composed of 18 images for evaluating model performance. The ground-truth quality for images was conducted by two radiologists (with longer than 10 years of experience) with disagreements resolved by a third independent expert rad and classified into one of five grades (grade 1, grade 2, grade 3, grade 4, and grade 5). Grade 5 is the best image quality. This reference standard complies with the situation assessed by the following quality criteria based on 5-point Likert scales [17–19] and is detailed in Table 1. The number of images from grade 1 to grade 5 was 21(23.6%), 23(25.8%), 12(13.5%), 16(18.0%), 17 (19.1%). Typical examples from each grade are shown in Fig. 3.

**Table 1:** A description of quality annotation standards

Quality label	Description
Grade 1	Inability to distinguish organs and tissue structures or obvious streak artifacts
Grade 2	The outline of the organ structure is not clear, or there are a few artifacts
Grade 3	The outline of the organ structure is relatively clear, and the lesions can be displayed
Grade 4	The outline of the organ structure is clear and free of artifacts
Grade 5	Anatomical contours are displayed clearly and without artifacts

### Image preprocessing

Image preprocessing included several operations and was conducted using Python (version 3.8.8) and MONAI (version 0.8.1) software. Firstly, Z -scores were used to normalize the scores, which had a normal distribution (99% of data had Z -scores between − 2 and 2). Secondly, all images were resampled to 128 × 128 × 256 pixels and 256 × 256 × 256 pixels, which were exported as input images. Thirdly, to increase the amount of training data, we used some transformations of the original training images [20]. Horizontal mirroring, rotations through random angles (± 10 degrees), gamma correction, and elastic deformation were used to produce new synthetic images [21, 22]. In addition, each PET image was resampled to isotropic spacing using linear interpolation to perform the model training or testing [23].

### Deep learning

Deep learning (DL) is a relatively new approach and is one branch of machine learning. DL has been ubiquitously applied in medical image analysis. The purpose of this study is to solve a five-class polyp classification problem. While, due to the very limited number of each grade, it is likely to cause bias and affect the accuracy of DL. Therefore, we merge data from five grades into two-class.

Finally, we generated four tasks, taking into account the resample of images and the quantity of each grade. Detailed information about each specific task in this study was described in Table 2.

**Table 2:** Grouping criteria for each task

Task	Resample	Class
Task1	128*128*256	1/2/3–4/5
Task2	256*256*256	1/2/3–4/5
Task3	128*128*256	1/2–3/4/5
Task4	256*256*256	1/2–3/4/5

### Dense convolutional network (DenseNet)

**Fig.4:**
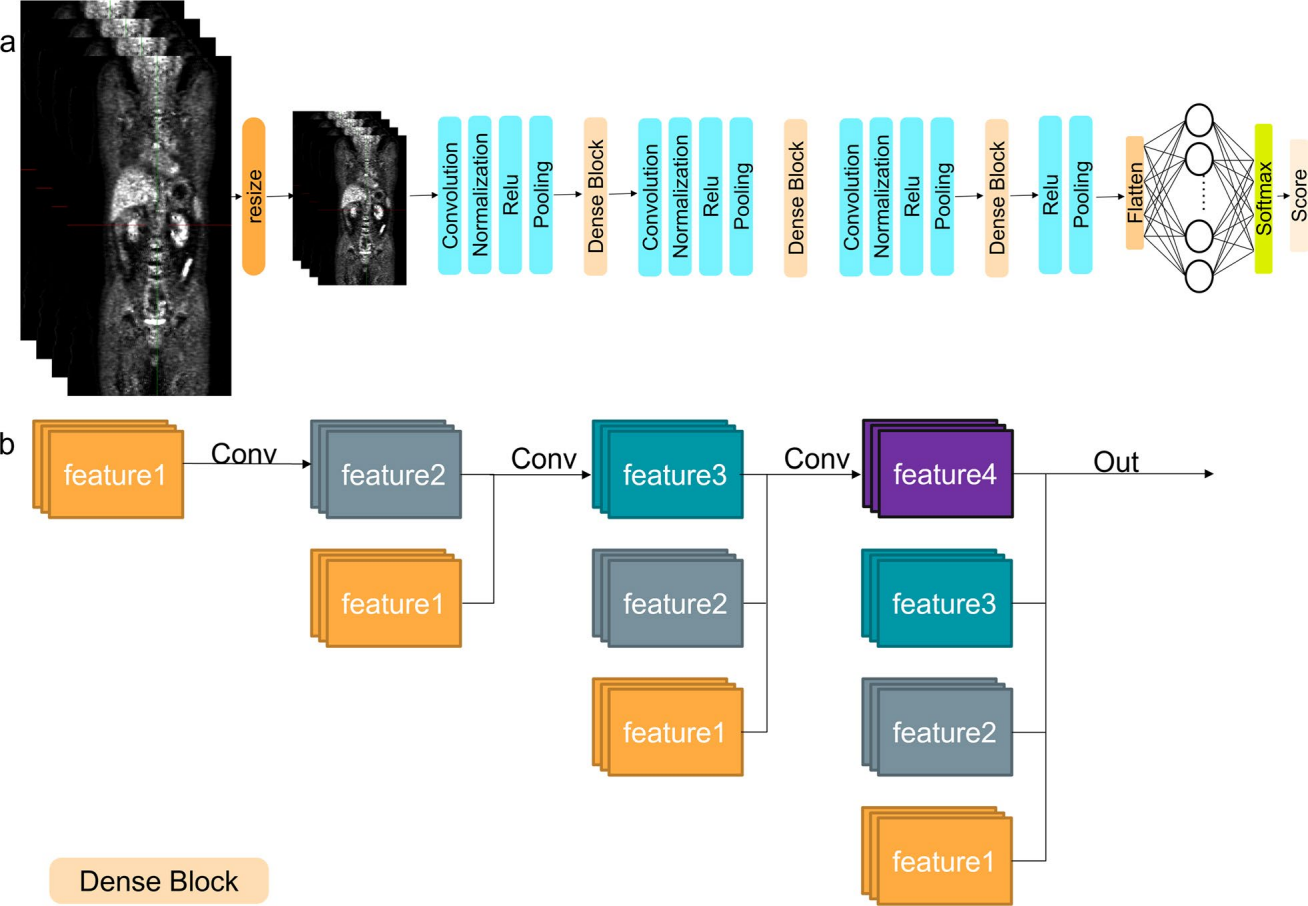
An illustration of the architecture of the deep learning-based model. Residual blocks for **a** DenseNet and **b** Dense Block

DenseNet connects each layer to every other layer in a feed-forward fashion. Thus, DenseNet has several compelling advantages: they alleviate the vanishing gradient problem, strengthen feature propagation, encourage feature reuse, and substantially reduce the number of parameters [24]. The overall architecture is shown in Fig. 4. A deep DenseNet with three dense blocks, where each dense block was multiple stacks of convolution, batch normalization, and ReLU activation layers. The layers between two adjacent blocks are referred to as transition layers and change feature map sizes via convolution and pooling.

### Model training

**Fig.5:**
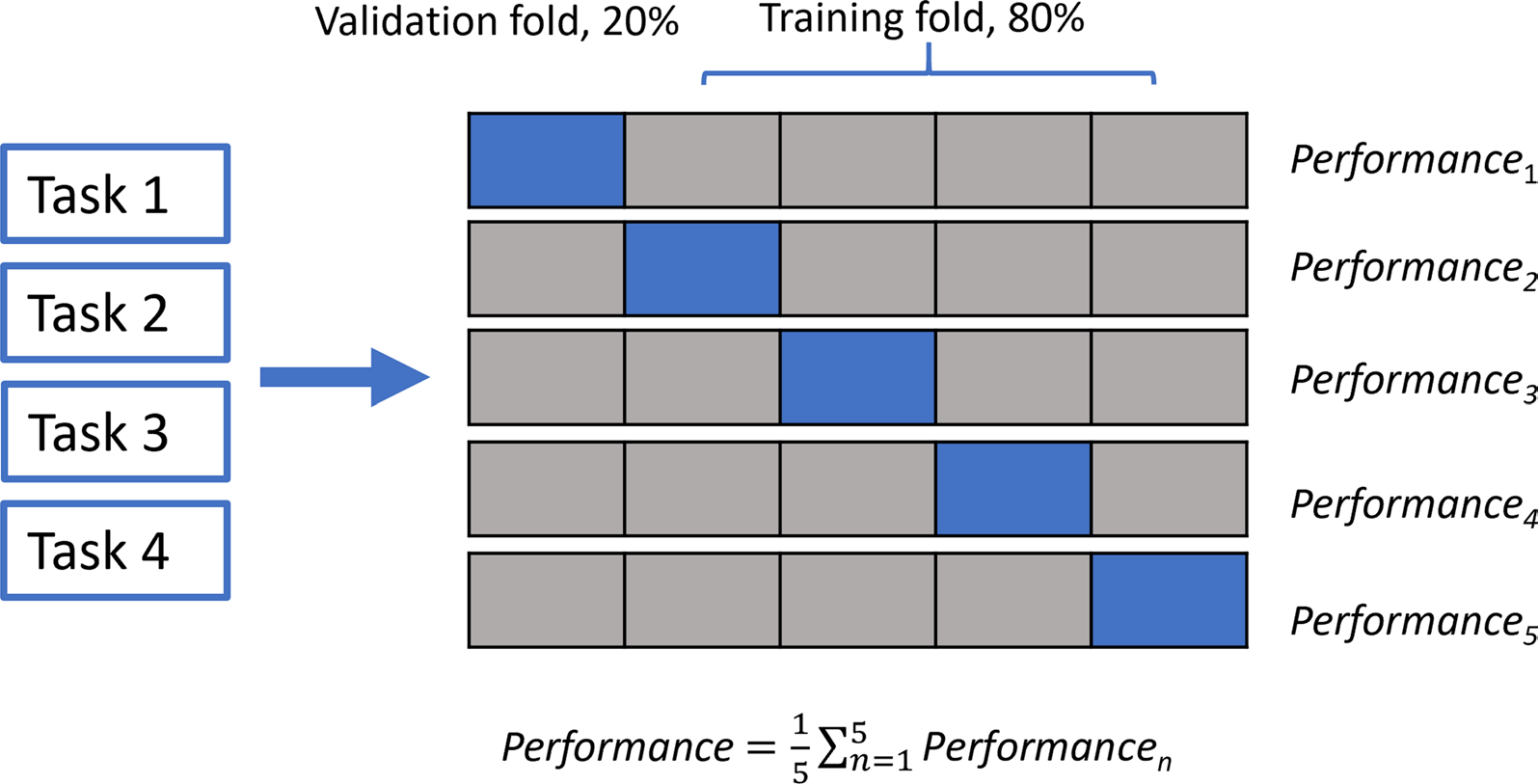
Training and validation workflow of each DL model for grade classification

This model also uses a 5 × 5 convolutional kernel, which has been shown to improve performance. The deep learning model was trained on the framework of Pytorch 1.10.1 version and an NVIDIA Quadro P3200 graphics card (NVIDIA, Santa Clara, CA) with 5 GB memory. A fivefold cross-validation with 80% training and 20% validation from each independent training was chosen to avoid bias in the data set (Additional file 1: Table S1 lists the distribution of data in training and validation sets for each fold of the cross-validation. Table 3 shows the image quality distribution for each of the different tasks using fivefold cross-validation in the training set and validation set.). The whole process is repeated 5 times such that all folds are used in the testing phase, and the average performance on the testing folds is computed as an unbiased estimate of the overall performance of the model, as shown in Fig. 5. In addition, the training loss function was binary cross-entropy. The optimizer was the Adam optimizer with a learning rate of ${10}^{-5}$. We set training iteration as 250 and run 1000 epochs on both the training set and testing set. We select polyLR as the learning rate, whose initial learning rate is 0.01.

**Table 3:** Distribution of image quality in training and validation sets for different tasks

Task	fold	Grade	Train (n)	Validation (n)
1&2	0	1/2/3	44	12
		4/5	27	6
	1	1/2/3	43	13
		4/5	28	5
	2	1/2/3	48	8
		4/5	23	10
	3	1/2/3	44	12
		4/5	27	6
	4	1/2/3	45	11
		4/5	27	6
3&4	0	1/2	36	8
		3/4/5	35	10
	1	1/2	34	10
		3/4/5	37	8
	2	1/2	37	7
		3/45	34	11
	3	1/2	35	9
		3/4/5	36	9
	4	1/2	24	10
		3/4/5	38	7

### Model performance

The automated classification in this study contains two classes, and we tested different multi-label classification algorithms. In this setting, the model classification performance is assessed in each class. Two-class model performance was determined by measuring the sensitivity (Sen), specificity (Spe), and accuracy (ACC) of all the introduced approaches. These parameters were calculated using Eqs. [25–28], where TP, TN, FP, and FN represent true positive, true negative, false positive, and false negative. Evaluation metrics were defined as follows:

1 $$\mathrm{a}\text{ccuracy} = \frac{\text{TP+TN}}{\text{TP+TN+FP+FN}}$$

2 $$\mathrm{s}\text{pecificity} = \frac{\text{TN}}{\text{TN+FP}}$$

3 $$\mathrm{s}\text{ensitivity} = \frac{\text{TP}}{\text{TP+FN}}$$

Performance was also evaluated via a receiver operating characteristic (ROC) curve. The area under the ROC Curve (AUC) is a general measure of the accuracy of a diagnostic test [29].

### PET QA report

An image QA tool was developed combining the DL-based image quality evaluation model. First, the PET/CT images were inputted into the QA tool, where the image statistics are gathered for statistics control, and registration is performed to examine the motion artifacts in the images. Next, the input images were evaluated using the deep learning PET image quality assessment model. Finally, the report will give the results of the image quality assessment (Good or Poor). The final output includes basic image information, scan and reconstruction parameters, typical instances of PET images, and deep learning score. The basic information in the output report includes patient information (such as age, gender, height, and weight), examination information (such as drug injected, time of injection, the dose of injection, time of injection), and equipment.

## Results

### Deep learning performance

The programming and computations were performed using a computer with a CPU of an Intel(R) Xeon(R) Silver 4110 @ 2.10 GHz processor and Tesla V100 GPU support. The proposed method was tested on PET images with four different tasks. The performance indexes in the evaluation of each task were reported based on the sensitivity, specificity, ACC, and AUC. We train all tasks by using the same training and testing strategy.

**Fig.6:**
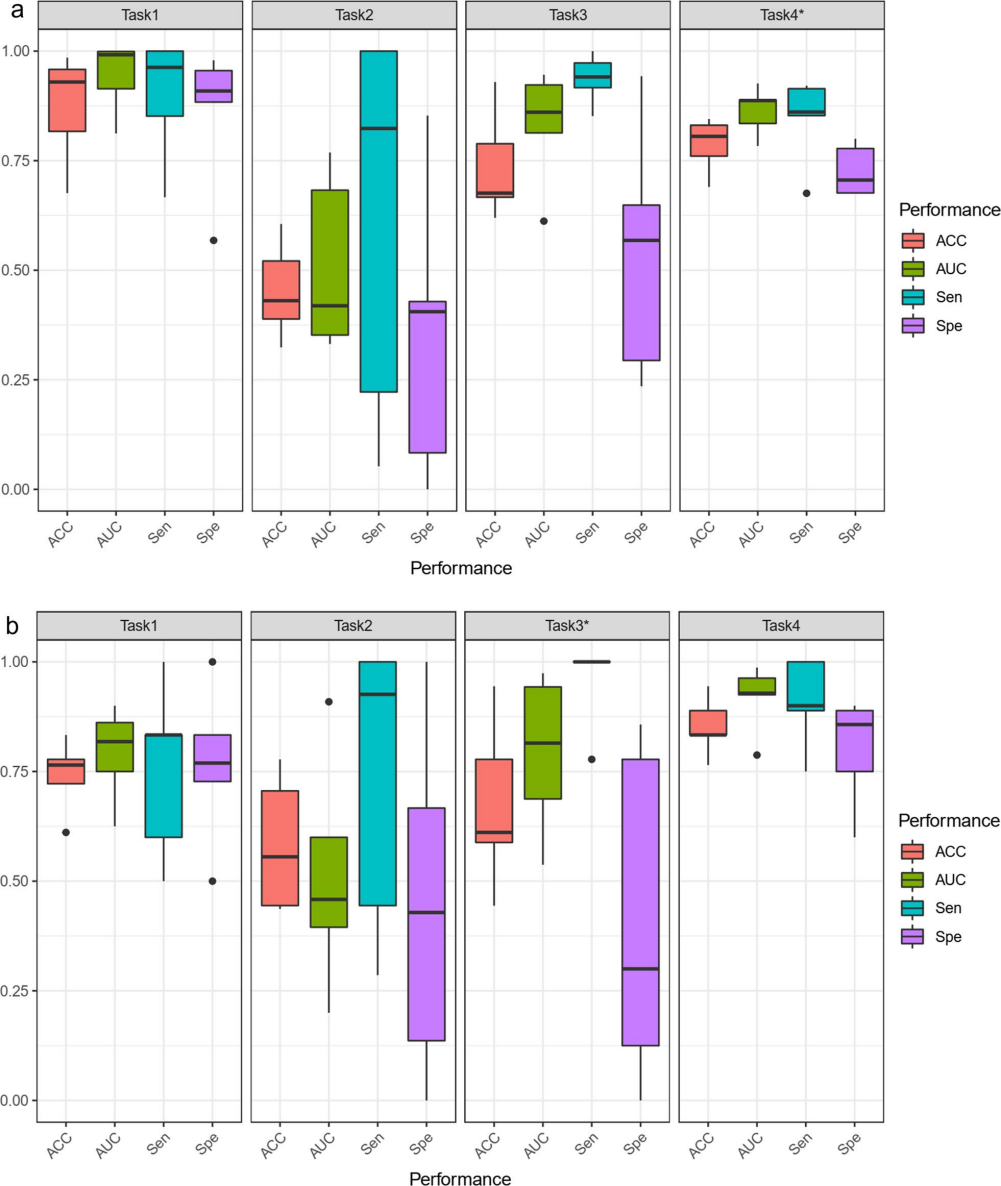
ACC, AUC, sensitivity and specificity results over fivefold cross validation experiment in training (**a**) and testing (**b**). For task1, in the train set, AUC = 0.94, ACC = 0.87, specificity = 0.86, and sensitivity = 0.90. In the test set, AUC = 0.79, ACC = 0.74, specificity = 0.75, and sensitivity = 0.77. For task 2, in the train set, AUC = 0.51, ACC = 0.45, specificity = 0.35, and sensitivity = 0.62. In the test set, AUC = 0.51, ACC = 0.58, specificity = 0.45, and sensitivity = 0.73. For task 3, in the train set, AUC = 0.83, ACC = 0.74, specificity = 0.54, and sensitivity = 0.95. In the test set, AUC = 0.79, ACC = 0.67, specificity = 0.42, and sensitivity = 0.95. For task 4, in the train set, AUC = 0.86, ACC = 0.77, specificity = 0.71, and sensitivity = 0.83. In the test set, AUC = 0.91, ACC = 0.85, specificity = 0.79, and sensitivity = 0.91

Figure 6 displays the ACC, AUC, sensitivity, and specificity for the four tasks over the fivefold cross-validation experiment in the training and testing data set. It can be seen that task 1 has the best overall performance (ACC = 0.87, Spe = 0.85, Sen = 0.90, AUC = 0.94), followed by task 4 in the training set. However, the specificity of task 4 was relatively poor at 0.71. Performances of task 3 were similar to task4, even the ACC, AUC, and sensitivity can outperform them on task 4. While the specificity for task 3 is far too low at 0.54. In terms of grouping conditions, tasks 3 and 4 both classify grades 1 and 2 as a group with poor image quality, and classify grades 3, 4, and 5 as a group with good image quality. Therefore, we think that perhaps this way of grouping images may have relatively low specificity. With the results of the validation group, we found that only the performance of task 4 can still maintain satisfactory results, and even improve the specificity to 0.8. Disappointingly, the performance of task 1 on the validation set dropped overall (ACC = 0.74, Spe = 0.77, Sen = 0.75, AUC = 0.79), and the model was relatively less stable. Based on the above results, we believe that task 4 is the model with the best comprehensive performance among the 4 tasks.

**Fig.7:**
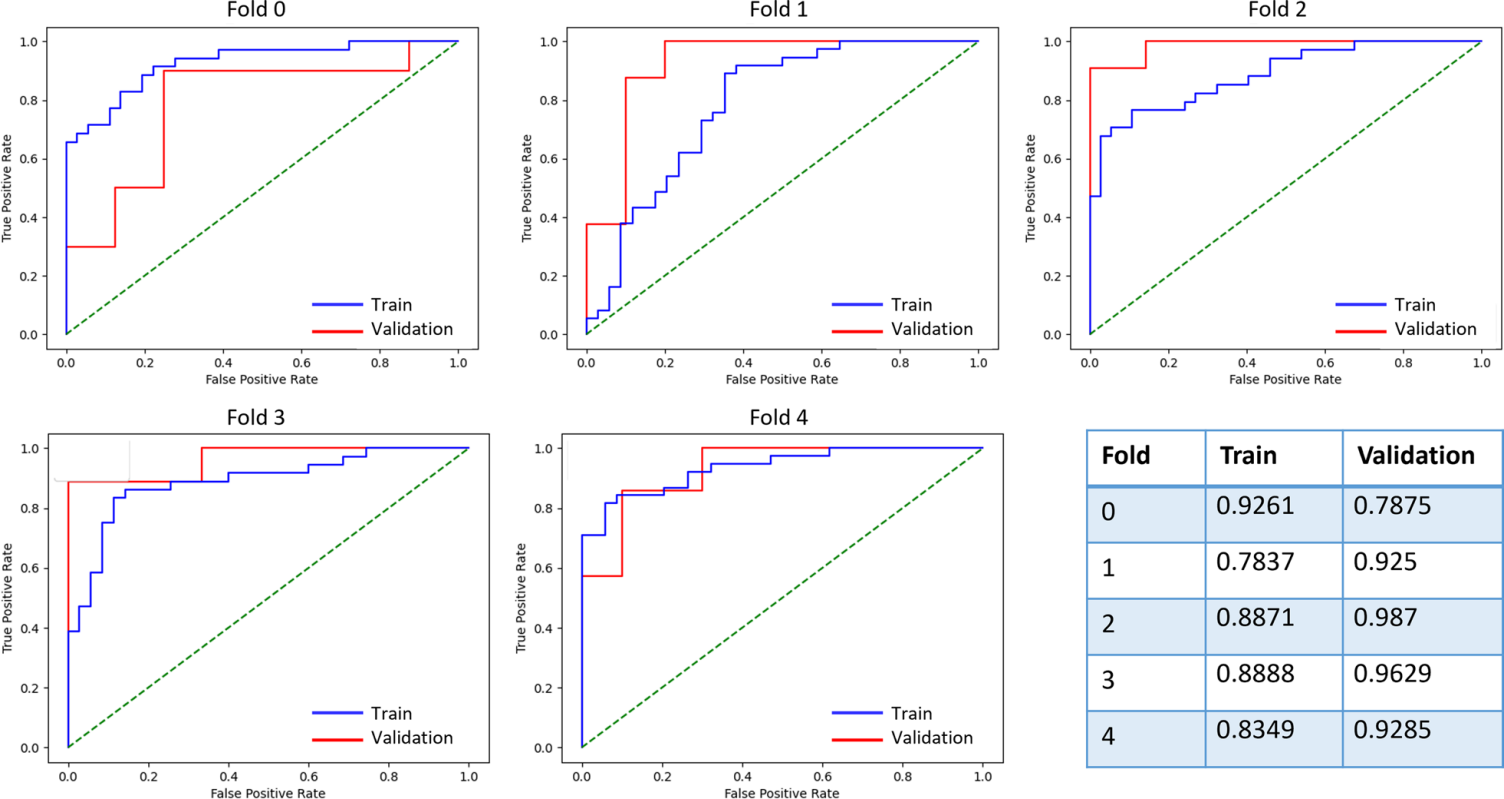
ROC curve for the training and validation set of task 4 for grade classification

**Fig.8:**
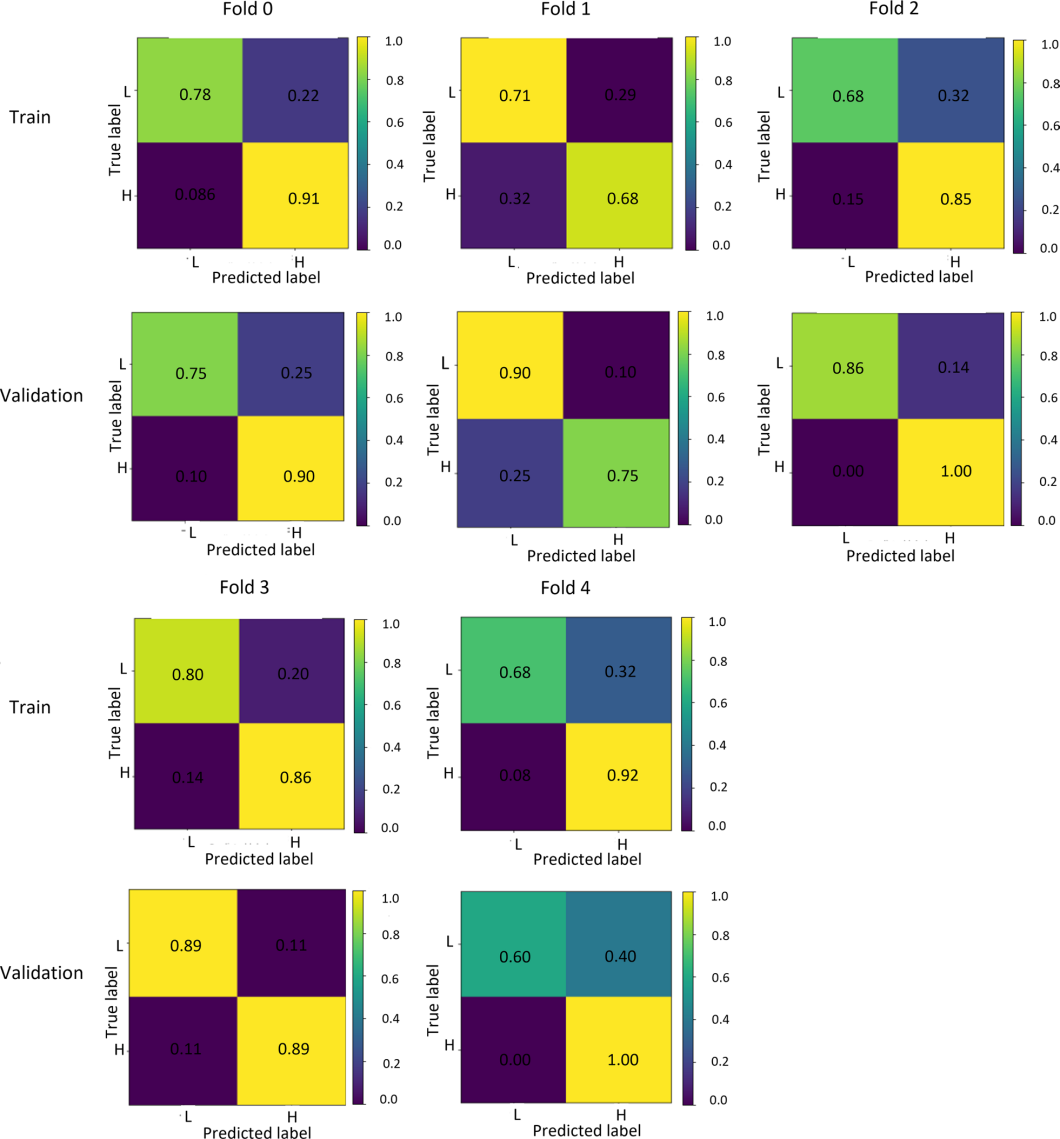
Confusion matrices and classification performance measures in task 4

Task 4 classifies the poor and optimal image quality successfully. Therefore, we describe here in detail the indicators of task 4, including all results in fivefold cross-validation. During five cross-fold validation, the best task was the task4 which reported an average AUC performance of 0.86, with a standard deviation of 0.06. The best-performing model of task 4 was used for subsequent analysis and had an AUC on the internal test set of 0.92. Task 4 showed a sensitivity of 0.91 and specificity of 0.80 for distinguishing between poor image quality (grade 1, grade 2) and optimal quality (grade 3, grade 4, grade 5) images. The overall accuracy of our classifier was calculated to be 0.85 for poor image quality images versus optimal quality images. The performance of each fold was reported in Table 4. The performance of the task4 was analyzed using ROC (Fig. 7), and the confusion is shown in Fig. 8.

**Table 4:** Performance of different folds for task 4

	Train				Validation			
Cross-fold	Accuracy	Specificity	Sensitivity	AUC	Accuracy	Specificity	Sensitivity	AUC
0	0.85	0.78	0.91	0.93	0.83	0.75	0.90	0.79
1	0.69	0.71	0.68	0.78	0.83	0.90	0.75	0.93
2	0.76	0.68	0.85	0.89	0.94	0.86	1.00	0.99
3	0.83	0.80	0.86	0.89	0.89	0.89	0.89	0.96
4	0.81	0.68	0.92	0.84	0.77	0.60	1.00	0.93
Average	0.79	0.73	0.85	0.86	0.85	0.80	0.91	0.92

AUC, area under the ROC Curve

### Case report

**Fig.9:**
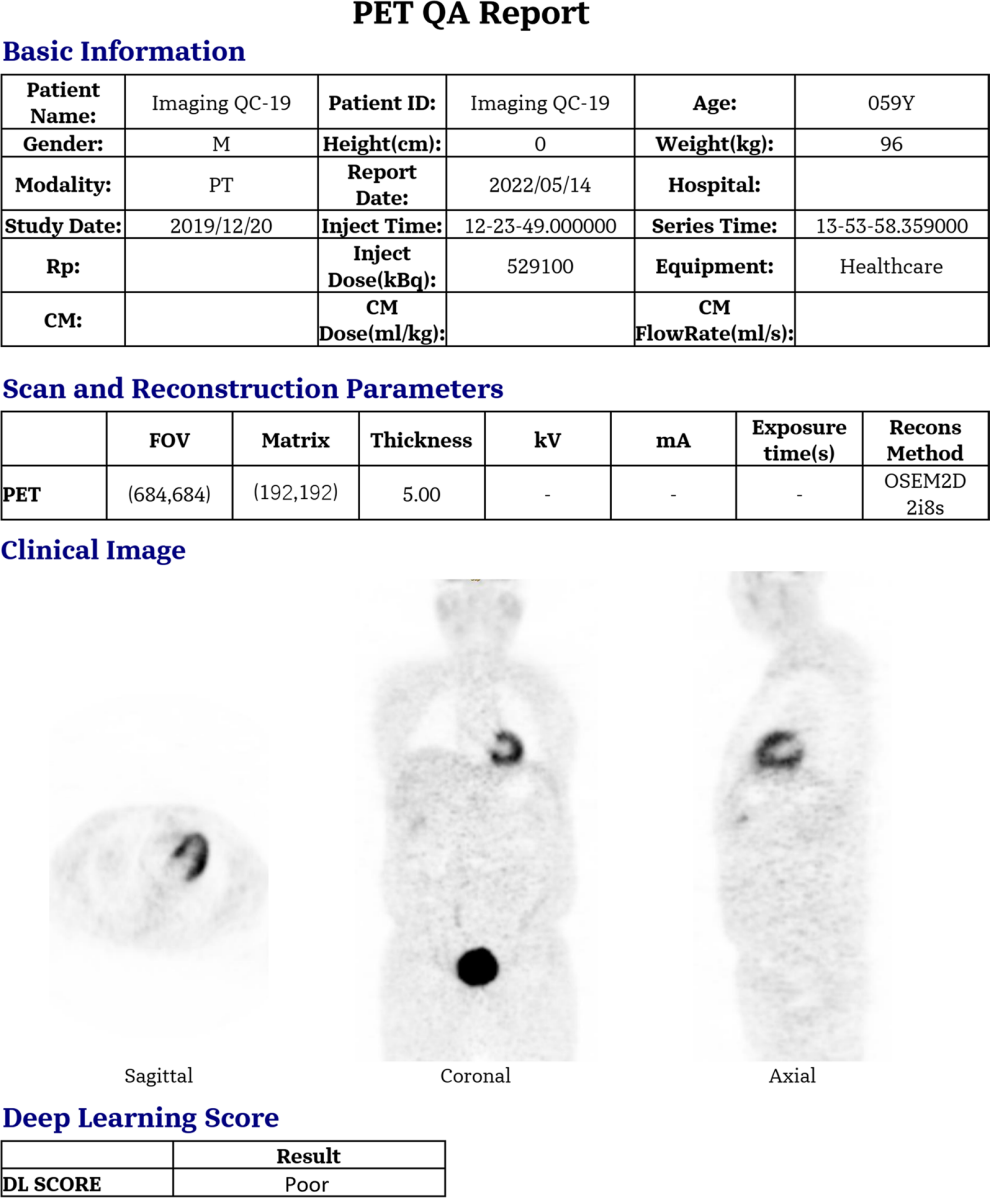
Case from PET QA Report where the poor quality of image assessed failed

The PET QA report can be obtained after the user inputs PET images. Typical classification results of fundus images are shown in Fig. 9, which shows the report of a poor quality of image generated by listmode data in reconstruction with one-third statistics. This reduction leads to an ultimately poor image quality assessed by a senior physician, which is consistent with the quality control report automatically generated by the quality rating system, confirming the usefulness of the PET image quality rating system. The typical instances of PET images and deep learning score in the case report can intuitively feedback the quality of images for the physicians who take charge of patient management. The scan needs to be repeated if the deep learning score shows poor result, such as Fig. 9. Moreover, the basic image information and scan and reconstruction parameters would guide the technicians to avoid the same failure in the repeated scan.

## Discussion

Since the quality of PET images is essential for further accurate data analysis, in this study, an architecture based on a deep DenseNet was evaluated for the assessment of image quality in PET images. We compared four tasks and verified their ability to assess the quality of PET images, respectively. Finally, we found that task 4 achieved the best performance in identifying poor image quality (grade 1, grade 2) versus optimal quality (grade 3, grade 4, grade 5) images where the images with grade 3 were also confirmed by three physicians and they were qualified in disease diagnosis. Task4 can provide rapid image classification and clinically relevant image features that can be used to provide feedback on image quality. Furthermore, the proposed deep learning model demonstrates the ability to classify images into two specified quality grades, which can be further applied to a quality control system to assist the automatic recognition of poor-quality images in the future. Thus, we developed a QA tool that uses the aforementioned deep learning method. The PET QA report obtained after inputting PET images can describe the information related to image quality in detail, which could help doctors to have a more comprehensive understanding of image quality.

Quality control of all medical images is critical, including PET images. To date, the research on the quality control of medical images mostly focuses on the ultrasound image, retinal images, ultra-widefield fluorescein angiography (UWFA), or OCT [10, 15, 16, 30–32]. There are few reports on quality control of PET images and implementing PET quality control platforms. Currently, PET image quality was assessed by manually drawing a sphere of hepar and comparing the mean liver intensity value across patients. However, this manual method consumes a lot of handling manpower and time. Herein, we propose a novel method for PET image quality assessment based on deep learning. Although only a few studies have addressed attempted to achieve PET image quality assessment based on deep learning, their inadequacies and shortcomings make them difficult to exert greater value in clinical applications. Elisabeth et al. [33] performed two CNNs trained to automatically identify EARL compliant images and separate if an image is meeting older or newer EARL standards. In their study, the two-dimensional image slices were used as input to the CNN and not the 3D information of the whole image. As we all know, the number of training data was enlarged by using 2D slices, thereby possibly increasing the classification performance of deep learning models. However, compared with 3D information of the image, 2D slices lose a lot of image-related information, which will lead to deviations in the accuracy of image quality assessment results. In order to avoid this situation, our study chose to input the 3D data into the model utilizing as much useful information as possible to complete the image quality assessment. In addition, we input the 3D whole-body PET image into the model, which can reflect the overall quality of the image compared with the local image. Thomas et al. [34] developed an automated pipeline for user-friendly and reproducible analysis of images with the aim of automating all processing steps up to the statistical analysis of measures derived from the final output images. Unfortunately, this study only analyzed brain images. In addition, the validation of each radiotracer accuracy was performed with differing ROI and using different methods for calculating parametric values. These differences mean that it is not possible to quantitatively compare their method accuracy for each radiotracer. This is enough to prove that better generalization can only be obtained by analyzing all aspects of the image rather than just a certain part. In further work, in addition to deep learning scoring, our study will comprehensively consider various factors and design a working flow of PET clinical imaging quality control to provide a step-by step evaluation of each key information point for physicians.

However, there were several important limitations of the present study that should be acknowledged. First, all whole-body PET images were obtained on a single PET-CT scanner. In the future, the reproducibility of this deep learning model should be tested across different scanners. Second, the data for this study were collected from the headless upper body. Therefore, PET data, including brain scans, will be considered in the follow-up investigation. Finally, the number of PET images in this study is relatively small, which limited the model’s ability to incorporate more data from either cohort. What’s more, there remains considerable data bias between different image quality grades. We did not validate our model in an external validation dataset. Additionly, image quality ratings are only made by three senior physicians, which would bias the results of assessment. To further refine our model and test the efficacy, we next will collaborate with clinics and other hospitals to collect more images to increase the number of PET images per grade, and also collaborate with more experienced physicians to make the rating more credible. In addition, when the number of images is large enough, we will try to perform five classifications and strive to achieve a more detailed division of image quality. We believe that concerted efforts in terms of data quantity and quality are needed in Densely Connected Convolutional Networks to make our deep learning model successful. Further, the value of this model for PET image quality assessment will be verified in clinical practice.

## Conclusion

In conclusion, this study highlights the feasibility of the assessment of image quality in PET images using a deep learning model. This method not only provides automated image selection for clinical PET image review but also provides feedback to image quality, which may assist with accelerating clinical research by reliably assessing image quality.

## Supplementary Information

**Additional file 1:** Distribution of image quality in training and validation sets for each fold.

## Data Availability

The data that support the findings of this study are available from the corresponding author upon reasonable request.

## Abbreviations

QA: Quality assessment
DL: Deep learning
PUMCH: Peking union medical college hospital
DenseNet: Dense convolutional network
ACC: Accuracy
ROC: Receiver operating characteristic curve
AUC: Area under the ROC Curve
PET/CT: Positron emission tomography/computed tomography
SUV: Standardized uptake value
SUL: Standardized uptake value normalized by lean body mass
CNN: Convolutional neural network
OCT: Optical coherence tomography
SNR: Signal-to-noise ratio
FDOPA: Fluorodopa
FDG: Fluorodeoxyglucose
OSEM: Ordered-subsets expectation maximization
FWHM: Full width at half maximum
TP: Ture positive
TN: Ture negative
FP: False positive
FN: False negative
Sen: Sensitivity
Spe: Specificity
UWFA: Ultra-widefield fluorescein angiography
