# Supplementary material for: Deep learning model for automatic image quality assessment in PET
Source: BMC Med Imaging. 2023 Jun 5;23:75. doi: 10.1186/s12880-023-01017-2 (PMC10243071; doi:10.1186/s12880-023-01017-2)
Supplement: Supplementary file 1 — Additional file 1: Distribution of image quality in training and validation sets for each fold. [file 12880_2023_1017_MOESM1_ESM.docx]

Table S1 Distribution of image quality in training and validation sets for each fold

| **Fold** | **Grade** | **Train** | **Validation** |
| --- | --- | --- | --- |
| 0 | 1 | 17 | 4 |
|  | 2 | 19 | 4 |
|  | 3 | 8 | 4 |
|  | 4 | 13 | 3 |
|  | 5 | 14 | 3 |
| 1 | 1 | 18 | 3 |
|  | 2 | 16 | 7 |
|  | 3 | 9 | 3 |
|  | 4 | 14 | 2 |
|  | 5 | 14 | 3 |
| 2 | 1 | 16 | 5 |
|  | 2 | 21 | 2 |
|  | 3 | 11 | 1 |
|  | 4 | 12 | 4 |
|  | 5 | 11 | 6 |
| 3 | 1 | 16 | 5 |
|  | 2 | 19 | 4 |
|  | 3 | 9 | 3 |
|  | 4 | 13 | 3 |
|  | 5 | 14 | 3 |
| 4 | 1 | 17 | 4 |
|  | 2 | 17 | 6 |
|  | 3 | 11 | 1 |
|  | 4 | 12 | 4 |
|  | 5 | 15 | 2 |
